# From blood development to disease: a paradigm for clinical translation

**DOI:** 10.1242/dmm.043661

**Published:** 2020-01-09

**Authors:** Monica J. Justice, Julija Hmeljak, Vijay G. Sankaran, Merav Socolovsky, Leonard I. Zon

**Keywords:** Blood disorders, Hematopoiesis, Model systems, Translational research

## Abstract

Translating basic research to the clinic is a primary aim of Disease Models & Mechanisms, and the recent successes in hematopoiesis research provide a blueprint of how fundamental biological research can provide solutions to important clinical problems. These advances were the main motivation for choosing hematopoiesis disorders as the focus of our inaugural meeting, ‘Blood Disorders: Models, Mechanisms and Therapies’, which was held in early October 2019. This Editorial discusses the reasons for and the challenges of interdisciplinary research in hematopoiesis, provides examples of how research in model systems is a key translational step towards effective treatments for blood disorders and summarizes what the community believes are the key exciting developments and challenges in this field.

## Abstract (precis)

**Summary:** This Editorial discusses how significant advances in the field of blood disorders have translated to successful treatments, offering a path to translation for other fields as well.

## Introduction

Sharing basic research findings with clinicians, and communicating clinical findings to basic researchers, is an important goal in efforts to improve human health. Researchers in the field of hematopoiesis (the development of blood cells) have made significant progress in this regard by taking discoveries from model organisms to the clinic and back again. The success of the field serves as a paradigm of the power of interdisciplinary research for providing functional insights into blood development and disease. The ability to deeply phenotype and manipulate blood cells in multiple model systems – including induced pluripotent stem cells (iPSCs), flies, fish and mice – has allowed for blood disorders to be among the first that are tractable to treatments. The treatment of severe combined immune deficiency (SCID) caused by adenosine deaminase deficiency paved the way for gene replacement therapies by placing the defective gene into vectors that could be transferred back into the patient through their blood stem cells (reviewed in Ferrua and Aiuti, 2017). Now, other blood diseases – including bleeding disorders, sickle cell disease, beta-thalassemia, immunologic diseases and a number of metabolic disorders – are being successfully treated by gene replacement or gene-editing therapies (reviewed in Dever and Porteus, 2017; The Lancet Haematology, 2019; High and Roncarolo, 2019). Although adapting gene therapy to other organ systems can be difficult, the field has learned a lot about safe vectors and delivery, the populations of cells to target and the approaches to deliver gene-editing tools *in vivo*. Similar approaches are now being used for the delivery of targeted therapies to the eye, brain and other organs. As the field continues to advance, safer methods of delivering reagents to correct precise mutations in genes through CRISPR/Cas9 gene editing or *ADAR* RNA editing may become available. Therefore, the advances in treatment strategies first developed in the hematopoietic system, including bone marrow transplantation, stem cell replacements, gene therapies and immunotherapies, have had a large impact on the treatment of non-blood disorders, such as cancer and infectious disease.

Translating basic research to the clinic is a primary aim of Disease Models & Mechanisms. Thus, we chose to showcase the advances in the field of hematopoiesis as a focus for our inaugural journal meeting, ‘Blood Disorders: Models, Mechanisms and Therapies’, which was held in Boston, MA, USA, in early October 2019. Building upon the recent translational successes and the exciting new developments discussed at the meeting, this Editorial discusses the reasons for and the key challenges of fostering interdisciplinary research in the field of hematopoiesis.

## Big data, small-animal models

**Fig. 1.**
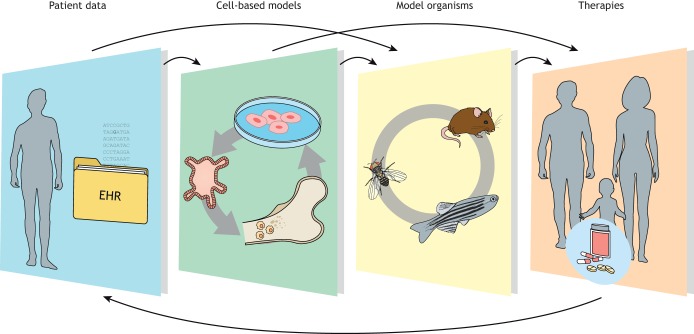
The field of blood disorders research is an excellent example of how integrating patient data with research in *in vitro* systems and model organisms can result in successful translation towards effective treatments. EHR, electronic health records.

New technologies have produced an outpouring of data at an unprecedented pace. Advanced computational methods, such as machine learning and scalable algorithmic approaches, are being developed to investigate questions related to human health and disease, integrating diverse data types, such as multiple omics data, or electronic health record phenotypes with genotype (Hatzopoulos, 2019). New sequencing technologies allow for whole-genome sequences to be produced at increasing sequencing depths, linking disease to genomic variation in unprecedented detail, but often raising questions of causality, which requires testing in model organisms (Fig. 1).

Many hematopoietic developmental and disease-related pathways are evolutionarily conserved, giving researchers in the blood field a plethora of model organisms. Research in *Drosophila* is aiding in the identification of key epigenetic and signaling pathways that become dysregulated in myeloproliferative neoplasms (Terriente-Félix et al., 2017; Bailetti et al., 2019). Improved genome annotation, ease of husbandry and the development of genome editing (see, for example, Prykhozhij and Berman, 2018), xenotransplantation (Parada-Kusz et al., 2018) and imaging tools have helped propel the zebrafish to the forefront of hematopoiesis modeling (reviewed in Konantz et al., 2019). Indeed, zebrafish models have informed the basis for malignant transformation in the hematopoietic niche (Zhang et al., 2019; Gjini et al., 2019). In parallel, isogenic cell line pairs that allow researchers to model hematopoiesis and interrogate the precise functional consequences of disease-causing mutations can now be produced using genome editing in patient-derived iPSCs (reviewed in Georgomanoli and Papapetrou, 2019).

Experimentation in cell or organ culture and non-mammalian models is typically fast and scalable, minimizing the cost of screening and proof-of-principle projects. However, before translating this knowledge to the clinic, it is essential to appropriately validate the results in more complex models, such as mice, which are particularly well suited for modeling hematopoietic disorders (see, for example, Belyea et al., 2018; Conway et al., 2018). In particular, mice have been instrumental in understanding the early embryonic origins of hematopoietic stem cells in the hemogenic endothelium (reviewed in Gao et al., 2018).

Despite their many advantages, *in vitro* systems currently complement, but cannot fully substitute, modeling in whole animals. With a scientific toolkit ranging from genomics to modeling, disease mutations are being discovered and analyzed at an astonishing pace. Therefore, moving from omics to function is likely to be a priority for ongoing technology development.

## Voices from the community

As the examples above illustrate, the hematopoiesis research community has many reasons for excitement, but progress also brings emerging challenges. The development of high-resolution approaches such as single-cell omics [RNA sequencing, assay for transposase-accessible chromatin using sequencing (ATAC-seq), DNA sequencing and methylation analysis] are giving us an unprecedented view of developmental pathways and of disease evolution, and have tremendous potential for translation to the clinic (reviewed in Strzelecka et al., 2018). Combined with cutting-edge imaging tools (e.g. intravital microscopy) and with genome editing, the hematopoiesis field is on the verge of a data explosion that will allow the formulation of new and sophisticated hypotheses to answer old questions. However, the large amounts of data produced with these tools can be difficult to digest, and require close cooperation between traditional (wet lab) and computational approaches. This interdisciplinary cooperation is still in its infancy and will need to become the norm.

Indeed, blood disorders research is a field in which many basic science insights are now beginning to merge with clinical and translational findings, giving researchers reason for excitement. This is particularly true in how studies of human genetics and biology dovetail nicely with many of the findings from model organisms. Although increasingly sophisticated and biologically relevant model systems, such as the zebrafish (reviewed in Konantz et al., 2019) and iPSC-derived *in vitro* models (reviewed in Georgomanoli and Papapetrou, 2019), provide many reasons for optimism, only select examples effectively relate findings in model organisms to human biology. This is a challenge that remains firmly in sight for researchers who use model systems. With the development of improved tools to study human biology, such as single-cell omics and lineage-tracing methods, the community can gain even deeper insights into hematopoiesis directly and develop accurate model systems for identifying therapeutic windows. With these advances will come the important challenge of comparative studies to ascertain both the similarities in various model systems and the key differences that could help to elucidate new biology.

Studies on the contribution of mutations in blood cells to disease provide lessons to the genomics community in understanding the relative contributions of germline and somatic mutations in both the coding and non-coding regions of the genome to disease presentation (reviewed in Rahman and Mansour, 2019), as well as natural variation in this process (reviewed in Bao et al., 2019). For example, in clonal hematopoiesis, a single cell may acquire a new mutation(s) that gives it a selective advantage to outcompete other cells, becoming the predominant clone, even if it carries traits that are detrimental. Sequencing of the blood cells reveals a single mutation, although progenitor cells reveal heterogeneity. Disorders such as cancer and bone marrow failure can be a result of clonal hematopoiesis and may have underlying germline genetic predisposition that is beginning to be uncovered.

A remaining issue is how to bring advances to the clinic faster. A model for a human disease can only be developed and studied singly, so producing a model for each becomes impossible for each of the huge number of germline and somatic mutations that are associated with disease. Moreover, for each disease, the pathway in which it acts provides information on variation in genetics and phenotype, yet such information takes time and money to produce. Some questions remain. Will cultured cells or organoids be able to replace or reduce the use of whole-organism models in these efforts? How will the field keep pace with the amount of data being generated, and how can funding agencies prioritize research efforts? Can comparative studies lead to the development of improved models, particularly with the availability of single-cell omics approaches?

## Conclusions

The examples from the literature summarized above also reflect the recurring themes that emerged from the ‘Blood Disorders’ meeting and remain a subject of continuous debate within the community. First, further research in developmental hematopoiesis is needed to deepen our understanding of the physiological developmental pathways that result in normal blood production, and to tackle pathological processes such as blood cancers, anemias, bone marrow failure disorders and even pulmonary hypertension. The comparative study of blood development across model organisms has contributed to our understanding, yet many gaps remain. Interestingly, as new models are developed, they continue to reveal new biology. Second, clonality and the mechanisms that allow a stem cell to become dominant in the hematopoietic niche remain important for understanding disease. However, conflicting data raise questions about the origin of dominant clones and their fate. Third, integrating insights from studies in humans with animal models, bringing together genomics and developmental biology, is key to spearheading a whole new interdisciplinary field. Fourth, epigenetic regulation is an additional confounding layer that can drive both normal and pathological processes. Undertaking any of these topics inevitably requires strong collaboration between experts from diverse fields, with the ultimate goal of translating this knowledge into the clinic. Designing a clinical trial – identifying targets, outcomes and biomarkers – is only a first step. Although the field of hematopoiesis may appear mature, it is so vast that new questions continue to arise. We look forward to exciting new developments.
